# Probable Levofloxacin-Induced Thrombocytopenia in a Patient Previously on Ciprofloxacin: A Case Report and Literature Review

**DOI:** 10.1155/2016/2860645

**Published:** 2016-01-14

**Authors:** A. Justine Landi, Robert Burkes

**Affiliations:** ^1^University of Louisville School of Medicine, Louisville, KY 40202, USA; ^2^University of Louisville Internal Medicine Residency Program, 550 S. Jackson Street, Louisville, KY 40202, USA

## Abstract

Drug-induced thrombocytopenia is a poorly understood, yet common phenomenon widely encountered in clinical practice. We present a case of suspected levofloxacin-induced thrombocytopenia, a rare side effect of a ubiquitous antibiotic, in a patient without similar effect to ciprofloxacin. This report builds upon other isolated case reports of fluoroquinolone-induced thrombocytopenia and demonstrates our algorithmic approach to the issue as well as a literature review pertaining to fluoroquinolone-induced thrombocytopenia.

## 1. Case

An 83-year-old white male with a history of type II diabetes mellitus, peripheral vascular disease, coronary artery disease, hypertension, and a diabetic foot infection with osteomyelitis of his right 5th metatarsal and proximal phalanx presented with decreased appetite and nausea. Bone biopsy one month prior to admission grew* Escherichia coli* and the patient was initially treated with 400 mg of oral ciprofloxacin twice daily for three weeks. The patient developed gastrointestinal intolerance and was switched to oral levofloxacin, 500 mg daily, one week prior to admission, which did not improve his gastrointestinal symptoms. The patient's review of systems was noncontributory aside from stated. The physical exam was notable for a positive probe-to-bone on a 1 cm-by-1 cm ulcer over the right medial malleolus and a 2 cm-by-1 cm right lateral foot ulcer along with decreased sensation over the feet bilaterally.

Laboratory results revealed a platelet count on admission of 128.0 × 10^9^/L (150–450 × 10^9^/L), down from a count of 480.0 × 10^9^/L one week prior to admission (and no previous episodes of thrombocytopenia) at the visit where ciprofloxacin was changed to levofloxacin. Peripheral smear showed no schistocytes, reduced peripheral platelet count without clumping, and a single anisocyte and ovalocyte per high-power field. The white blood cell count was 5.6 × 10^9^/L (4.5–11.0 × 10^9^/L) with 81.5% neutrophils, hemoglobin 11.0 g/dL (13.5–17.5 g/dL) with a mean corpuscular volume of 83.3 fl, and red cell distribution width of 20%. Blood chemistries showed sodium of 140 mEq/L (135–145 mEq/L), potassium of 4.0 mEq/L (3.5–5.0 mEq/L), chloride of 106 mEq/L (95–105 mEq/L), bicarbonate of 23 mEq/L (22–29 mEq/L), blood urea nitrogen of 49 mg/dL (7–18 mg/dL), creatinine of 2.2 mg/dL (0.6–1.2 mg/dL) with baseline appearing to be 1.2 mg/dL, and blood glucose of 83 mg/dL (70–115 mg/dL). Haptoglobin, lactate dehydrogenase, vitamin B12, and folate were normal. Other than the addition of the antibiotic, there was no recent changes in his medication.

The differential diagnosis for the patient's thrombocytopenia consisted of dilutional effect secondary to fluid resuscitation, heparin-induced thrombocytopenia (HIT), myelodysplastic syndrome, splenic sequestration, and other drug-induced thrombocytopenias. During the hospital course, the hemoglobin reached a nadir of 9.5 g/d, but stabilized as the platelet count continued to trend downward ruling out dilutional effect. An absence of schistocytes on peripheral smear made intravascular consumption less likely. An ultrasound with no sign of hepatomegaly or splenomegaly decreased the likelihood of splenic sequestration. HIT was unlikely due to the fact that the patient reported no recent prior exposure to unfractionated or low molecular weight heparin, and this was confirmed by a negative HIT antibody panel. Myelodysplastic syndrome was thought to be unlikely per hematology consultants. Because of this, our concern for drug-induced thrombocytopenia increased.

**Figure 1:**
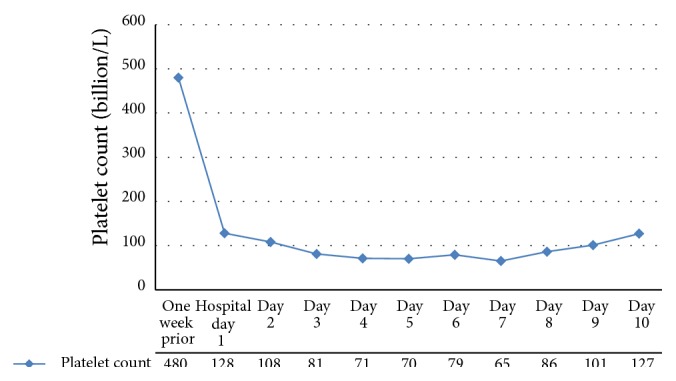
Case of patient's platelet trend showing initial point one week prior to admission where ciprofloxacin was changed to levofloxacin, as well as trend throughout hospital stay. Levofloxacin was changed to cefepime on hospital day 4.

Hematology consultants introduced the idea that the patient's thrombocytopenia could be a result of a drug reaction to the fluoroquinolones. The antibiotic regimen was changed from levofloxacin to cefepime on hospital day 4 and the platelets gradually rose to 127 × 10^9^/L at discharge (Figure 1). At three-week postdischarge follow-up, the patients platelets were 391 × 10^9^/L and would remain near this level at subsequent follow-up visits.

## 2. Denouement

We diagnosed this patient with probable fluoroquinolone-induced thrombocytopenia based on decrease in platelet count after the introduction of levofloxacin and the improvement in platelet count after the removal of this agent.

Drug-induced thrombocytopenia (DIT) is a relatively common disorder marked by a moderate-to-severe thrombocytopenia and possible signs of spontaneous bleeding that range from ecchymosis and petechia to mucosal bleeding to life-threatening spontaneous intracranial hemorrhage [1]. There are several mechanisms for this phenomenon. Certain drugs, particularly chemotherapeutics, may cause a predictable or idiopathic myelosuppression that becomes evident at variable times after administration. Some medications can cause a peripheral cytopenia, wherein the life spans of circulating blood components are shortened with no effect on the bone marrow [2]. Further, a rapid deceleration in the production of platelets upon administration of an offending drug may be indicative of an autoimmune process [3].

A literature review on fluoroquinolone-induced thrombocytopenia shows recent case reports with patient outcomes ranging from asymptomatic reduction in platelet count to severe thrombocytopenia associated with diffuse microhemorrhage and death [4–7]. These respective cases cover intravenous fluoroquinolones. The time to nadir of observed thrombocytopenia after drug administration ranged from 12 hours [4] to a week [5] after first dose of fluoroquinolone in two cases concerning intravenous ciprofloxacin. The lowest reported platelet count was 2 × 10^9^/L in a case involving alatrofloxacin (three days after first dose) [6]. In all three cases with reported survival, several days passed before platelets returned to a level above the lower limit of normal, similar to the trajectory of recovery seen in the presented case [4–6].

In a case reporting death, there were no bone marrow abnormalities nor microangiopathy noted on autopsy. Examination revealed diffuse petechial hemorrhages suggesting that death was caused exclusively by peripheral thrombocytopenia without associated bone marrow suppression or thrombotic thrombocytopenic purpura [7].

One study reports an in-depth investigation of the development of glycoprotein IIb/GIIIa directed antibodies in the serum of their patient with fluoroquinolone-induced thrombocytopenia [4]. The authors drew the hypothesis that the similarities in the structure of the central ring of fluoroquinolones and quinine, a known cause of autoimmune thrombocytopenia, are responsible for this reaction [3, 4]. In vitro analysis of humans and mice with quinine-induced immune thrombocytopenia shows the presence of anti-glycoprotein IIb/IIIa IgG that increases in binding affinity for its antigen after interaction with soluble quinine molecules [8, 9]. Despite a mechanism being suggested for ciprofloxacin, there are no reports in these antibodies cross-reacting in the presence of other fluoroquinolones.

The described phenomenon of a particular fluoroquinolone causing thrombocytopenia despite tolerance to another fluoroquinolone has not been described. However, anaphylaxis has been reported in a patient after levofloxacin exposure who was otherwise able to tolerate full dose oral garenoxacin after skin patch testing showed a reaction to the former but not the latter [10]. Likewise, ciprofloxacin tolerance has been described in a patient who had an anaphylactic reaction to levofloxacin [11]. A small trial of patient with hypersensitivity to a single fluoroquinolone showed unpredictable and low rates of cross-reactivity between fluoroquinolones but concluded that the authors had been the first to show a cross-reactivity between levofloxacin and other fluoroquinolones [12]. It remains unclear whether this IgE-mediated hypersensitivity reaction is analogous in its molecular target of the fluoroquinolone center ring as the IgG reaction proposed for fluoroquinolone induced thrombocytopenia.

Certain criteria suggest possible DIT [13]. DIT becomes more likely if there was no other change in medication prior to the initiation of the inciting agent or rechallenge with that agent causes another fall in platelet count [3]. This was organized into a systematic approach by George et al. in 1998 [13] through the following criteria:

1. Therapy with the suspected drug was instituted before thrombocytopenia and platelet count recovered upon stopping medication.
2. The suspected medication was the only new medication instituted.
3. No other causes of low platelets were identified.
4. Reexposure resulted in concomitant drop in platelets.

Meeting all four criteria gives a “definite” diagnosis, while meeting criteria (1), (2), and (3) gives a “probable” diagnosis and meeting criteria (1) gives a “possible” diagnosis, and the diagnosis is “unlikely” if criteria (1) is not met.

Several laboratory tests have been developed to evaluate the presence of markers of drug-induced antibodies, but these suffer from poor solubility of certain drugs, need for specific patient cells for testing, and inability to test drug metabolites for causative factor [1]. Specific testing is difficult and not widely available, with the exception of heparin [3].

Recovery from DIT is variable and based on the amount of bone marrow destruction caused by the particular agent. It is recommended that a bone marrow evaluation should be undertaken in a patient that requires blood-product administration for their thrombocytopenia or myelosuppression. The presence of precursor cells in the bone marrow aspirate is a promising sign for a more rapid recovery of cell counts than absence of these cells [3]. Resolution of peripheral cell counts is expected with withdrawal of the offending agent [2, 3].

Upon determining sensitivity to a particular agent, the agent should be removed from the patient's treatment and should be listed as a drug intolerance. The immunogenic response to a particular agent is usually quite specific and similar drugs may be instituted in treatment without increased risk for DIT [2, 3]. Likewise, the above-mentioned case demonstrates that tolerance to a particular drug in one class does not correlate with tolerance to the entire class of drugs.

## 3. Conclusion

The above case demonstrates fluoroquinolones as a suspected medication causing DIT and also illustrates our algorithmic approach to this issue. Further, tolerance to one medication in a class does not confer tolerance to the entire class, as described above. It is imperative to rule out other causes of thrombocytopenia and be able to employ diagnostic criteria for DIT, especially for nonchemotherapeutic or heparin agents, to come to an accurate diagnosis.
